# *Anopheles coluzzii* from Sakassou, Central Côte d’Ivoire show aggravated resistance to pyrethroids and organophosphate but are fully susceptible to clothianidin

**DOI:** 10.1186/s13071-026-07296-2

**Published:** 2026-03-10

**Authors:** Louise G. Bellai, Benjamin G. Koudou, Jürg Utzinger, Pie Müller, Constant A. V. Edi

**Affiliations:** 1 Centre Suisse de Recherche Scientifiques en Côte d’Ivoire, Abidjan, Côte d’Ivoire; 2 Swiss Tropical and Public Health Institute, Allschwil, Switzerland; 3 University of Basel, Basel, Switzerland; 4 Laboratoire d’Entomologie, Unité de Formation et de Recherche Sciences de La Nature, Université Nangui Abrogoua, Abidjan, Côte d’Ivoire

**Keywords:** Côte d’Ivoire, Cytochrome P450 monooxygenases, Indoor residual spraying, Insecticide resistance, Insecticide-treated nets, Knockdown resistance, Malaria control

## Abstract

**Background:**

Insecticide-based interventions, including insecticide-treated nets (ITNs) and indoor residual spraying (IRS), are central to malaria vector control in sub-Saharan Africa. In Côte d’Ivoire, increasing insecticide resistance in the key malaria vector, *Anopheles gambiae* sensu lato (*An. gambiae* s.l.), has been reported across the country, potentially compromising the current ITN-based control strategy. To assess the feasibility of supplementing control efforts with IRS using clothianidin as an alternative insecticide in areas with high malaria prevalence, we have examined the intensity and molecular mechanisms of insecticide resistance in wild *An. gambiae* s.l. populations with a focus on pyrethroids and the neonicotinoid clothianidin.

**Methods:**

Using the World Health Organization (WHO) insecticide susceptibility test, we assessed the intensity of insecticide resistance in 2- to 5-day-old female *An.* *gambiae* s.l. mosquitoes from Sakassou, Central Côte d’Ivoire, collected locally at the larval stage, against the pyrethroids alpha-cypermethrin, deltamethrin and permethrin, the neonicotinoid clothianidin and the organophosphate pirimiphos-methyl. To characterise the mechanisms underlying insecticide resistance, we conducted synergist assays using piperonyl butoxide (PBO) in combination with pyrethroids. Additionally, we performed diagnostic PCR to determine the *An. gambiae* s.l. sibling species and to characterise resistance mechanisms targeting the knockdown resistance (*kdr*) markers L995F and L995S and the insensitive acetylcholinesterase (*Ace-1*^*R*^) G280S resistance marker. We also compared expression levels of the key cytochrome P450 monooxygenases (P450s) CYP6M2, CYP6P3, CYP6P4 and CYP6P5 between field-collected mosquitoes and those from a laboratory insecticide-susceptible colony.

**Results:**

The diagnostic PCR identified all *An. gambiae* s.l. specimens that yielded a positive result as *Anopheles coluzzii.* *Anopheles coluzzii* individuals showed resistance to all pyrethroids tested, with very low mortality rates for alpha-cypermethrin and deltamethrin (range: 0% to 4%) and permethrin (range: 0% to 1%). In contrast, mortality rates against the organophosphate pirimiphos-methyl ranged from 52% to 97%. However, all mosquitoes remained fully susceptible to the neonicotinoid clothianidin (100% mortality). Pre-exposure to PBO increased the mortality rates following exposure to pyrethroids but did not restore susceptibility completely, with mortality rates of between 4.9% and 30.7% for alpha-cypermethrin, between 5.4% and 68.2% for deltamethrin and between 1.9% and 18.7% for permethrin. Among the four key resistance genes assessed in pyrethroid resistance, only the CYP6M2 gene showed significant overexpression (fold change 2.1, *p*-value = 0.025) in the field-sampled *An. coluzzii* compared to the susceptible laboratory colony. Taken together, our results suggest the involvement of a metabolic resistance mechanism. In addition to metabolic resistance, we detected all three target-site resistance alleles (i.e. *kdr*-L995F, *kdr*-L995S and *Ace.1*-G280S), with *kdr*-L995F predominating, with allelic frequencies ranging from 53% to 71% across years.

**Conclusions:**

*Anopheles coluzzii* mosquitoes from Sakassou show high levels of resistance to pyrethroids but remain susceptible to clothianidin, indicating that clothianidin-based IRS may serve as an effective complementary strategy for malaria control.

**Graphical Abstract:**

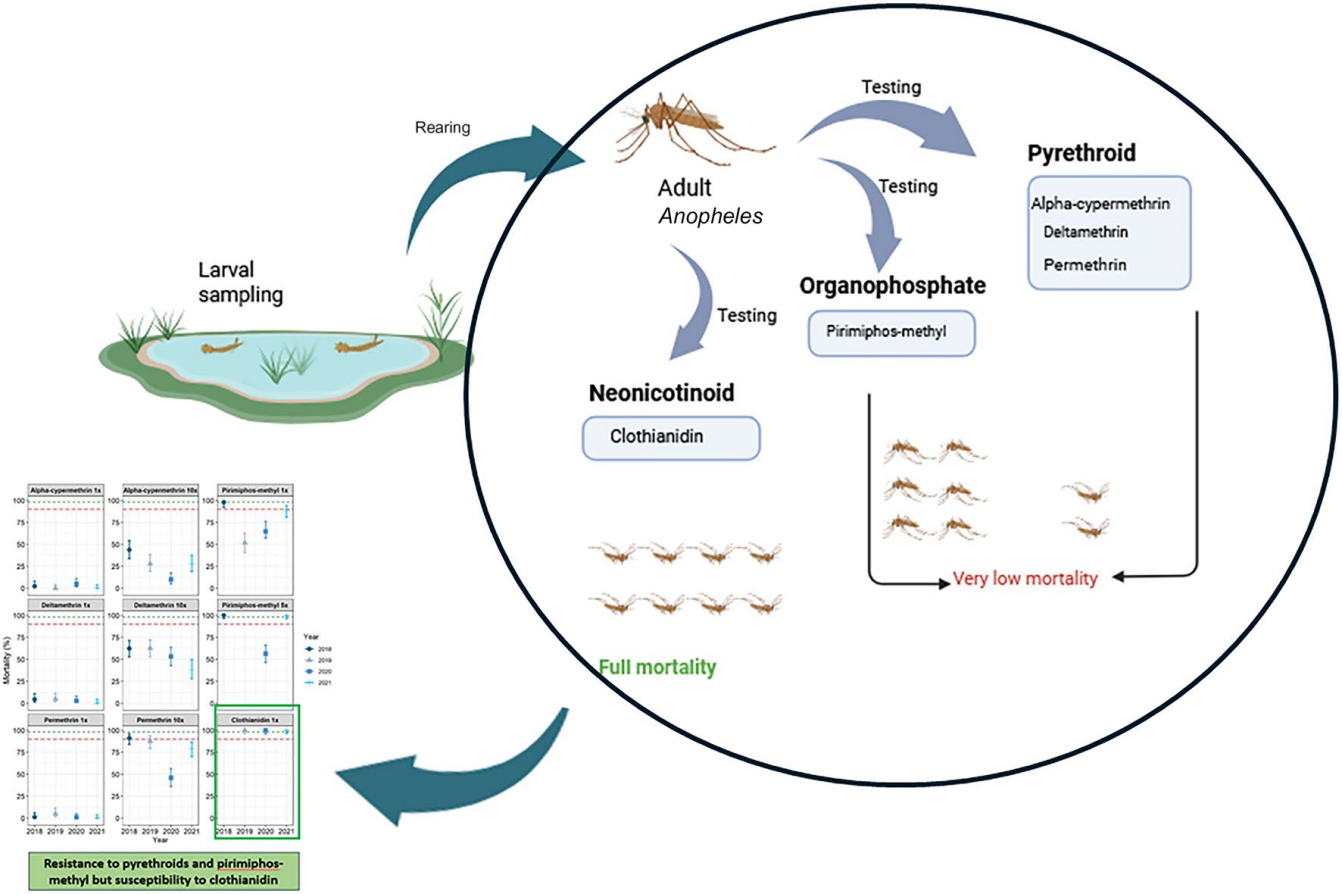

## Background

Vector control based on insecticide-treated nets (ITNs) and indoor residual spraying (IRS) has contributed substantially to the control and elimination of malaria and saved millions of human lives since the beginning of the new millennium [1]. However, progress in malaria control has stalled in recent years, which has partially been attributed to rising resistance to insecticides in *Anopheles* mosquito vectors [2].

In 2019, at the time when the study presented here was launched, a World Health Organization (WHO) report showed that Côte d’Ivoire was among the five countries with the highest malaria prevalence in sub-Saharan Africa [3]. In Côte d’Ivoire, vector control relies on the distribution of pyrethroid-treated nets, with an estimated 14 million nets distributed every 3 years [4]. Despite these efforts, malaria remains endemic across Côte d’Ivoire [5]. The incidence of malaria in Sakassou, a health district located in the central part of Côte d’Ivoire, is among the highest in the country [4], despite a net coverage exceeding 80% [6]. Between 2011 and 2017, the National Malaria Control Programme distributed two brands of deltamethrin-impregnated nets—Permanet 2.0 (Vestergaard; Lausanne, Switzerland) and Yorkool LN (Tianjin Yorkool International Trading; Tianjin, China)—with at least one ITN provided to every household [7].

One possible explanation for the high malaria incidence in Sakassou, despite high net coverage, is the development of resistance to pyrethroids in the key malaria vectors, namely the *Anopheles gambiae* sensu lato (*An. gambiae *s.l.) species complex [8, 9]. These mosquitoes have been shown to be resistant against a wide range of insecticides, including pyrethroids, carbamates, organophosphates and organochlorines [10–12]. Worryingly, insecticide resistance in *An. gambiae* s.l. is increasing in Côte d’Ivoire [10, 11, 13]. Despite this suspicion of increasing pyrethroid-resistant *An. gambiae* s.l. mosquitoes in Sakassou, the actual situation remains unknown.

Due to the continuous spread of insecticide resistance in *Anopheles* vectors, new compounds and formulations are being developed, or repurposed, to restore efficacy of insecticide-based vector control interventions. Recently, WHO has prequalified Fludora® Fusion (Bayer Crop Science; Monheim, Germany), an IRS product for mosquito control that combines deltamethrin and clothianidin, two insecticides with unrelated mechanisms of action [14]. Deltamethrin is a pyrethroid and works by binding to the voltage-gated sodium channel in the insect nerve cells, causing these channels to stay open longer, resulting in continuous nerve stimulation. This overstimulation leads to paralysis and ultimately the death of the insect [15]. Clothianidin is a neonicotinoid and acts on the central nervous system of insects as an agonist by stimulating nicotinic acetylcholine receptors, targeting the same receptor site and activating post-synaptic acetylcholine receptors [16–18]. Fludora® Fusion has been demonstrated to be effective against mosquitoes with resistance to pyrethroids, carbamates and organophosphates [19, 20], thereby representing a promising intervention in areas where mosquitoes show a high resistance to pyrethroids.

In this study, we evaluated the insecticide resistance levels of *An. gambiae* s.l. to three pyrethroids (i.e. alpha-cypermethrin, deltamethrin and permethrin), an organophosphate (i.e. pirimiphos-methyl) and a neonicotinoid (i.e. clothianidin) to assess whether clothianidin-based IRS could potentially complement existing ITNs distributed in Sakassou. Additionally, we investigated potential molecular mechanisms associated with the observed resistance phenotypes in individual mosquitoes using PCR diagnostics for target-site resistance and overexpression of detoxification enzymes.

## Methods

### Study site

**Fig. 1:**
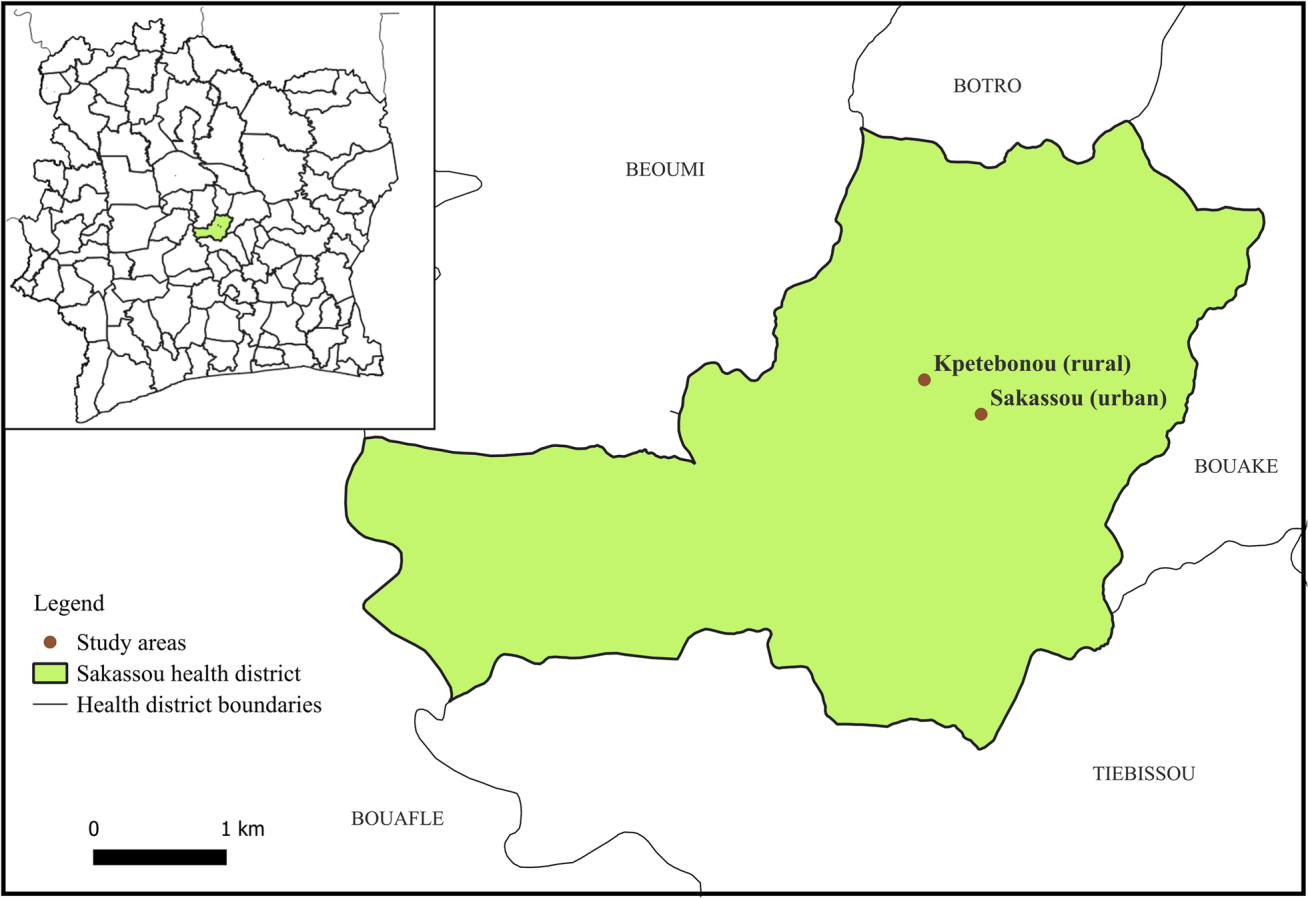
Map of Sakassou health district in Central Côte d’Ivoire showing larval sampling areas. The base map is from Open Street Map (OSM) [23]. The map in the inlet depicts the Sakassou health district (green area), created using the Global Administrative Areas (GADM) website [24]

The study was carried out in the Sakassou health district located in the pre-forested savannah zone in Central Côte d’Ivoire (Fig. 1). The local climate is tropical with two rainy seasons (March–June and September–October) and two dry seasons (July–August and November–February). The annual average rainfall is 899 mm and the annual average temperature is 26.1 °C [21]. The main agricultural activities of the local population in Sakassou are irrigated rice farming with water from the Bandama River followed by vegetable cropping. In the Sakassou health district, malaria is the first cause of medical consultation, and malaria transmission occurs all year round with the peak transmission period occurring during the rainy seasons in April and September [6, 22]. Sakassou town (7.45462°N, 5.29263°W), an urban area, and Kpetebonou village (7.47921°N, 5.32931°W), a rural area, were selected for mosquito larvae collection (Fig. 1). Kpetebonou village is located in a lowland area devoted to intensive irrigated rice farming.

### Mosquito sampling

*Anopheles* mosquito larvae were collected over a 4-year period (2018–2021), always in June and July. *Anopheles gambiae* s.l. larvae exploit small reservoirs with standing, clear and sunlit water bodies and we therefore focused on these types of water bodies for larvae collection. Using the traditional dipping method [25], we collected larvae and pupae from various habitats that met these criteria, such as rice fields, water pools and vegetable gardens. We then transferred the larvae and pupae to a field laboratory, where we pooled them together and reared them to adults. Larvae were reared in transparent plastic trays using water from the breeding sites and fed on TetraMin fish food (Tetra GmbH; Melle, Germany) until adult emergence. A thermometer and a hygrometer were used to ensure that the rearing room was maintained at a temperature and relative humidity range of 25–28 °C and 70–80% respectively. The larvae were reared at a relatively higher temperature (room temperature between 25 °C and 28 °C), while adults were placed in cooler conditions (air conditioner, 24–26 °C). Emerging adults were provided with 10% sugar solution ad libitum until 2–5 days post emergence. Adult mosquitoes were identified using the morphological key of Coetzee et al. [26], and those identified as *An. gambiae* s.l. were used in the WHO insecticide susceptibility bioassays [27].

### Bioassays

We conducted insecticide susceptibility tube tests following WHO guidelines [27], and susceptibility was tested against the diagnostic dose of three pyrethroids (i.e. alpha-cypermethrin 0.05%, deltamethrin 0.05%, permethrin 0.75%), an organophosphate (i.e. pirimiphos-methyl 0.25%) and a neonicotinoid (i.e. clothianidin 2%). Initially, we tested all insecticides at the diagnostic concentration (1×). All mosquitoes survived exposure to the diagnostic concentrations of the pyrethroids and the organophosphate; therefore, we subsequently ran intensity assays at fivefold (5×) and tenfold (10×) the diagnostic concentrations, as per WHO protocol [27]. Insecticide-treated filter papers were obtained from the University Sains of Malaysia. To obtain filter papers impregnated with the diagnostic dosage of clothianidin 2%, we followed the standard operating procedure established by the Vector Link team [28] by diluting 264 mg of Sumishield WG50 (Sumitomo Chemical; Osaka, Japan) in 20 ml of distilled water. We used four Whatman no. 1 filter papers (Whatman, GE HealthCare Life Sciences [Cytiva]; Marlborough, MA, USA) of 12 cm × 15 cm each and we distributed 2 ml of solution on each of the filter papers, corresponding to a concentration of 13.2 mg/active ingredient (ai) of clothianidin per filter paper. Untreated filter papers impregnated with 20 ml of distilled water served as the control for testing with clothianidin.

We tested the insecticides against an *An.* *gambiae* sensu stricto (*An.* *gambiae* s.s.) Kisumu strain colony that was susceptible to check for their quality prior to using them in the assays with the field-sampled specimens.

For each insecticide, concentration and year, we exposed 80–100 female mosquitoes to the treatment in batches of 20–25 mosquitoes per tube. We also exposed mosquitoes to control filter papers that were only treated with the carrier oil. While the WHO guidelines recommend that 50 mosquitoes be used in each control, we only exposed a single batch of 20–25 mosquitoes due to the limited availability of test mosquitoes. In the tests involving the three pyrethroids, we also conducted synergist tests using 4% piperonyl butoxide (PBO) [27] and pre-exposed mosquitoes to PBO for 1 h before exposure to the diagnostic concentrations of the pyrethroids.

In each assay, we scored the number of mosquitoes that: (i) were knocked down during the 60-min exposure to the insecticide and (ii) were dead 24 h post exposure. In the assays with clothianidin, we continued scoring mortality at 24-h intervals for up to 7 days post exposure. Scores for delayed mortality were considered to be valid if the 24-h mortality in the unexposed controls run in parallel was < 20%. After exposure to the insecticides, or to the control filter paper, we transferred the mosquitoes back to the holding tubes and provided them with 10% sucrose solution ad libitum by placing sucrose-soaked pieces of cotton at the top of the holding tubes.

### Sample preservation and nucleic acid extraction

Prior to DNA extraction, we stored the mosquitoes from the insecticide susceptibility tests in 1.5-ml Eppendorf tubes (1 mosquito per tube) with silica gel until DNA extraction. For DNA extraction, we removed one or two legs and the wings from each specimen and followed the protocol described by Collins et al. [29]. For each annual survey, we extracted DNA from a randomly selected subset of specimens, sampled across all bioassays.

For the gene expression analysis, we preserved mosquitoes collected in 2021 from the pyrethroid insecticide susceptibility assays that were still alive 24 h post-exposure as well as those from the unexposed control mosquitoes in RNA*later* (Ambion; Austin, TX, USA) and stored them at −80 °C until RNA extraction. For total RNA extraction, we used the Low Input Quick Amp Labelling Kit (Agilent Technologies Stratagene; Santa Clara, CA, USA), following the protocol described in Haoues et al. [30]. RNA was extracted from three batches of 10 mosquitoes from each group (i.e. 30 mosquitoes surviving post-exposure pyrethroid insecticide testing and 30 unexposed mosquitoes from control tubes) together with three biological replicates of 10 mosquitoes from an insecticide-susceptible *An. gambiae* s.s. Kisumu laboratory colony.

### *Anopheles gambiae* s.l. species identification

Due to delays in the shipment of PCR reagents in 2021, we ran no target-site resistance assays for that year; only the gene expression assay was run in 2021 as reagents for these assays were available. Likewise, we performed *An. gambiae* s.l. species identification with the available reagents. To discriminate between *An.* *gambiae* s.s. and *An. coluzzii*, we followed the short-intercept element (SINE200) PCR protocol described by Santolamazza et al. [31]. To perform the PCR assay, we used two primers: 6.1a (5′-TCGCCTTAGACCTTGCGTTA-3′) and 6.1b (5′-CGCTTCAAGAATTCGAGATAC-3′). The final reaction volume of 25 µl contained a mix of 12.5 µl OneTaq Quick-Load 2× Master Mix (New England Biolabs; Ipswich, MA, USA), 5.5 µl nuclease-free water, 0.5 µl dimethyl sulphoxide (DMSO), 2 µl bovine serum albumin (BSA), 1 µl of each primer, 0.5 µl MgCl_2_ and 2 µl DNA template. The amplification of the DNA took place in a thermocycler, with the following cycling programme: 35 cycles of 94 °C for 5 min, 95 °C for 25 s and 54 °C for 30 s for 1 min; with a final step at 72 °C for 10 min. We ran the resulting PCR products in an ethidium-stained 1.5% agarose gel, visualised with a UV illuminator.

### Resistance marker genotyping

For the detection of target-site resistance alleles, including both the L995F and L995S knockdown resistance (*kdr*) mutations (L995F/S-*kdr*) and the G280S acetylcholinesterase 1 resistance (*Ace-1*^*R*^) mutation, we performed PCR analysis. The Taqman assay described in Bass et al. [32] was used to identify the L995F/S-*kdr*, and the Taqman assay described in Bass et al. [33] was used to identify the G280S *Ace-1*^*R*^ mutation. For all study years except 2020, we screened 50 specimens from the pyrethroid bioassay testing and 25 specimens from the organophosphate testing (composed of dead and alive); in 2020, we tested 20, 23 and 12 specimens for L995F-*kdr*, L995S-*kdr* and G280S-*Ace-1*^*R*^ mutations, respectively, due to shortage of reagents during the COVID-19 pandemic.

All reactions were carried out in an Agilent Technologies Stratagene MX3005 qPCR thermocycler (Agilent Technologies). For each reaction, we used a final reaction volume of 10 µl containing 1 µl of template DNA and 9 µl of total master mix. The mix contained 3.875 µl DNase-free water, 5 µl SensiMix (250 nM) (Bioline; Memphis, TN, USA) and 0.125 µl of specific primers and probes for each of the *kdr* alleles L995F and L995S and the *Ace-1*^*R*^ G280S allele (1 µM each). The specific probes contained the FAM and HEX fluorochromes. The FAM-labelled probe detects the mutant allele, while the HEX-labelled probe detects the wild type of the susceptible allele. PCR assays were run as follows: 1 cycle of 10 min at 95 °C, followed by 40 cycles of 10 s at 95 °C and 45 s at 60 °C.

### Insecticide resistance gene expression

We assessed the expression patterns of genes coding for four potentially pyrethroid-detoxifying cytochromes P450s (P450s), including CYP6M2, CYP6P3, CYP6P4 and CYP6P5, which were previously implicated in pyrethroid resistance in Côte d’Ivoire [34, 35], using reverse transcriptase-quantitative PCR (RT-qPCR). We followed the protocol described in Kwiatkowska et al. [36] using the Brilliant III Ultra-Fast SYBR Green qPCR Master Mix (Agilent Technologies) and ran the assays in a BioRad CFX qPCR system (BioRad Technologies; Singapore, Republic of Singapore). The qPCR primers were obtained from the PMI annual report published in 2021 [28]. For each reaction, 1 µg of total extracted RNA from each of the three biological replicates from the mosquitoes that survived the WHO pyrethroid susceptibility testing, the unexposed control and the laboratory reference strain Kisumu served as template to synthesise the complementary desoxyribonucleic acid (cDNA) using Superscript III (Invitrogen, Thermo Fisher Scientific; Waltham, MA, USA) with oligo-dT20 and RNase H, following the manufacturer’s instructions. A serial dilution of cDNA was used to establish standard curves for each gene to assess PCR efficiency and quantify differences between samples.

### Data analysis and interpretation

All data analysis was performed using R software version 4.4.1 [37]. For the formal statistical tests, we set the level of significance to *α* = 0.05. For the phenotypic insecticide susceptibility assays, we calculated the mean percentages of dead mosquitoes and their 95% confidence intervals (CIs) using a generalised linear model (GLM) with a binomial distribution and a logit link function. We used the WHO criteria to classify the mosquito population’s resistance status based on the mean percentages [27]: (i) if < 90% of the mosquitoes survived the WHO discriminating concentration of an insecticide, they were deemed to be resistant; (ii) if the mortality rate was between 90% and 98%, the mosquito population was suspected to be resistant; and (iii) if the mortality rate was ≥ 98%, the mosquito population was deemed to be susceptible.

We hypothesised that pre-exposure to PBO would increase the susceptibility to pyrethroids in pyrethroid-resistant mosquitoes due to metabolic resistance mechanisms. Hence, we tested the effect of pre-exposure to PBO using GLMs with a binomial distribution and a logit link function with ‘PBO’ (i.e. with vs. without PBO) and ‘year’ as explanatory terms. We also included, “year”, as a term to determine whether there was a difference across years. The changes in mortality rates are reported as odds ratios (ORs). Additional GLMs were used to model the effect of year on mortality rates for the other insecticides.

We determined the allelic frequencies of the *kdr* alleles L995F and L995S, as well the frequency of the G280S *Ace-1*^*R*^ mutation. To assess whether these alleles showed potential signs of selection, we compared the observed genotypic frequencies with the expected frequencies under Hardy–Weinberg equilibrium (HWE), using the exact test procedure from the ‘Hardy–Weinberg’ package [38] implemented in R software.

The level of gene expression was assessed using the relative fold-change (FC) measurement, which allows comparison of the level of expression between a candidate gene in the resistant and susceptible laboratory colony. The relative expression and the FC of each target gene were calculated according to the 2-ΔΔCT method, incorporating PCR efficiency after normalisation with the housekeeping genes RSP7 (ribosomal protein S7, AGAP010592) and actin 5C (AGAP000651) [39]. In practice, we normalised output raw CT-values obtained from the real-time PCR system on efficiency correction and then calculated the average ΔCT value (CT target − CT control) across genes.

## Results

### Phenotypic insecticide resistance

**Fig. 2:**
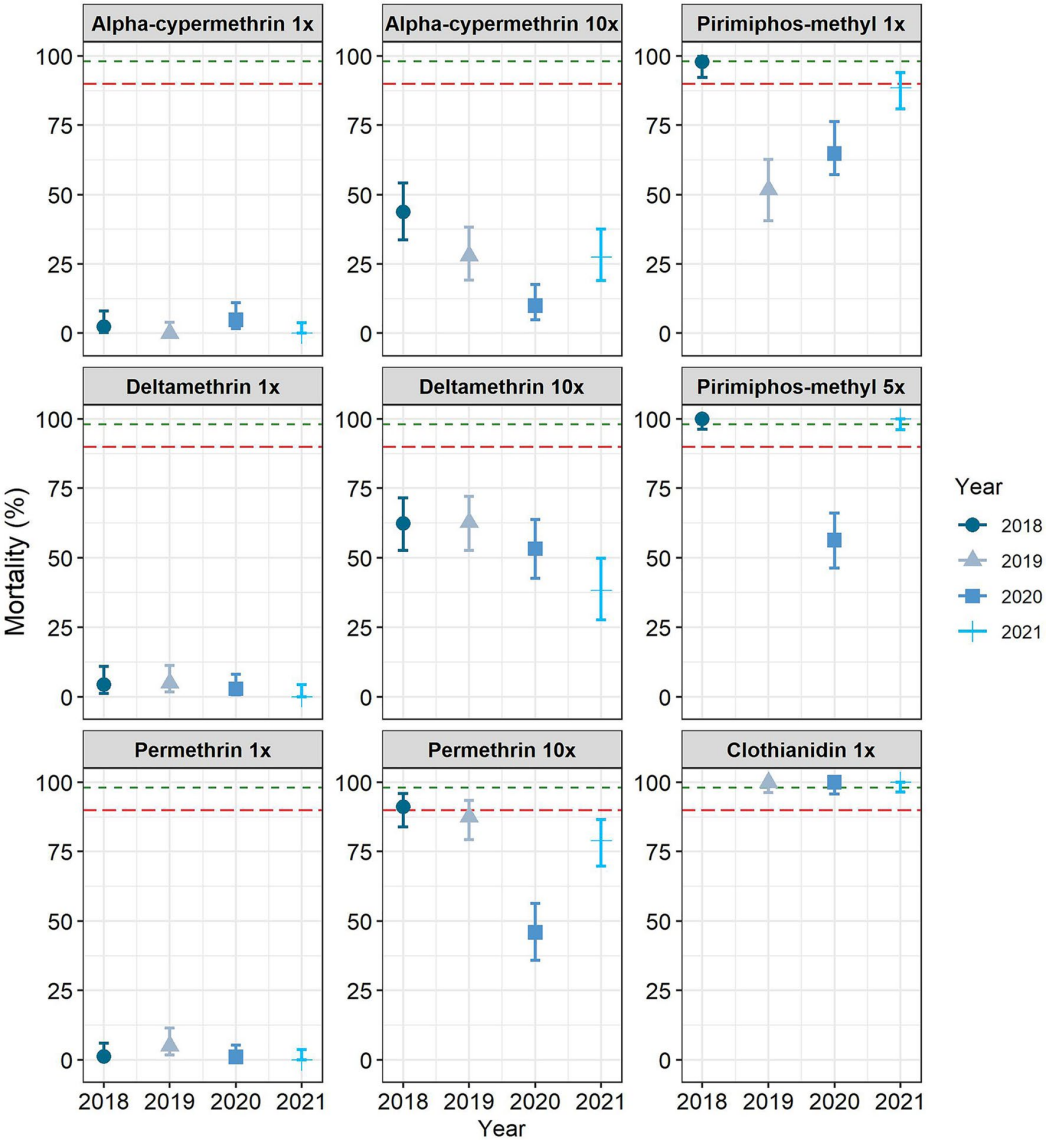
Phenotypic insecticide resistance in *Anopheles gambiae* sensu lato from Sakassou, Central Côte d’Ivoire, assessed using the WHO insecticide susceptibility assay. The symbols represent mean mortality rates and the vertical lines represent the 95% confidence intervals around the means. The horizontal red dashed line represents the 90% insecticide resistance threshold. The horizontal green dashed line indicates the suspected threshold for insecticide resistance. 1×, Diagnostic dosage; 5×, fivefold diagnostic dosage; 10×, tenfold diagnostic dosage

The field-collected *An. gambiae* s.l. showed high levels of resistance to all three pyrethroids (alpha-cypermethrin, deltamethrin and permethrin) as well as resistance to pirimiphos-methyl, while they were susceptible to clothianidin (Fig. 2). Mortality rates were very low following exposure to the diagnostic concentration (1×) of all three pyrethroids, and exposure to tenfold (10×) the diagnostic dosage did not reach the 100% mortality: alpha-cypermethrin 1× (mortality 0–4%), alpha-cypermethrin 10× (mortality 5.3–43%); deltamethrin 1× (mortality 0–4%), deltamethrin 10× (39.8–63.5%); permethrin 1× (mortality 0–1%) and permethrin 10× (mortality 45.8–91.3%). Mortality rates following exposure to the organophosphate were low: pirimiphos-methyl 1× (mortality 51.9–97.7%), pirimiphos-methyl 5× (mortality 57.2–100%). There were no clear patterns of decrease in mortality over the study period. Mortality rates of 100% were recorded with clothianidin over the study period.

**Fig. 3:**
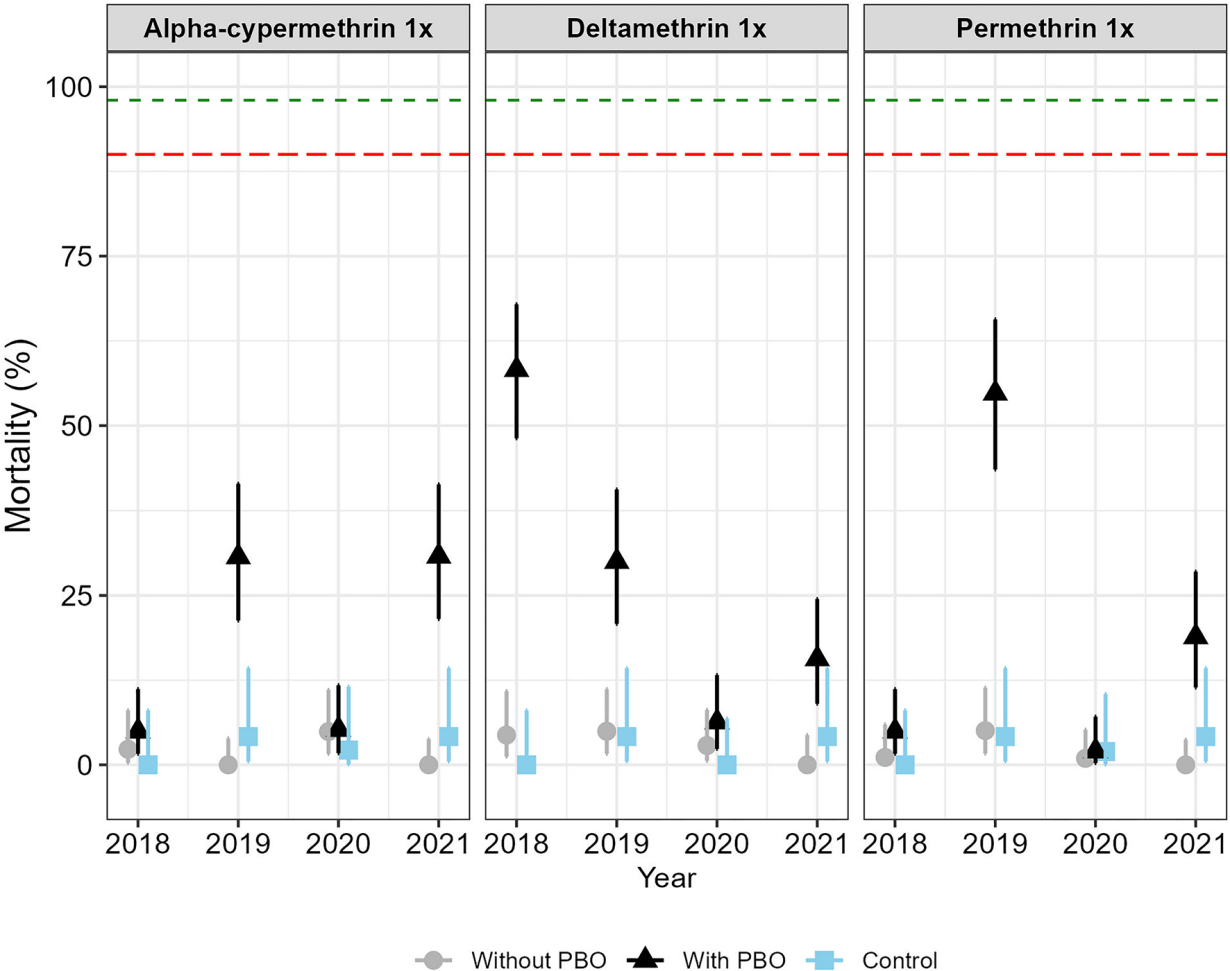
WHO synergist-insecticide bioassays comparing mortality rates following exposure to pyrethroids in *Anopheles gambiae* sensu lato from Sakassou, Central Côte d’Ivoire with and without pre-exposure to PBO. The bioassays included exposure to diagnostic pyrethroid dosage with and without pre-exposure to 4% PBO. The symbols represent the mean mortality rates, and the vertical lines represent the 95% confidence intervals around the means as estimated by generalised linear models. The horizontal red dashed line represents the 90% insecticide resistance threshold. The horizontal green dashed line indicates the suspected threshold for resistance. 1×, Discriminating (diagnostic) dose of the pyrethroids. PBO, Piperonyl butoxide

Pre-exposure to PBO increased susceptibility to the pyrethroids in most instances (Fig. 3; Table 1). However, susceptibility was not restored completely, with mortality rates ranging from 4.9% to 30.7% following exposure to alpha-cypermethrin, from 5.4% to 68.2% following exposure to deltamethrin and from 1.9% to 18.7% following exposure to permethrin. In some cases, pre-exposure to PBO did not change the susceptibility levels to pyrethroids.

**Table 1:** Increase in mortality rates upon pyrethroid exposure following pre-exposure to piperonyl butoxide in *Anopheles gambiae* sensu lato from Sakassou, Central Côte d’Ivoire

Insecticide	Year	With vs without PBO pre-exposure^a^	*p*-value
Alpha-cypermethrin 0.05%	2018	2.21	0.35
	2019	6.31	< 0.001
	2020	0.48	0.49
	2021	6.7	< 0.001
Deltamethrin 0.05%	2018	30.4	< 0.001
	2019	0.27	0.08
	2020	0.08	0.004
	2021	6.3	< 0.001
Permethrin 0.75%	2018	4.7	0.16
	2019	4.9	0.19
	2020	0.46	0.64
	2021	1.6	< 0.001

The bioassays included exposure to the diagnostic dosage (1×) of pyrethroids with and without pre-exposure to 4% piperonyl butoxide (PBO)

^a^Presented as the odds ratio

These findings suggest that other resistance mechanisms might be involved. Susceptibility to the pyrethroids following pre-exposure to PBO varied during the 4 years of the study (Table 1; Fig. 3).

### Molecular species identification

Among the 200 *An. gambiae* s.l. specimens screened, the diagnostic PCR identified 196 mosquitoes as *An. coluzzii*; the remaining four specimens could not be identified by PCR. These results suggest that the mosquito species tested in the insecticide resistance and molecular assays were all, or at least predominantly, *An. coluzzii*.

### Insecticide target-site mutations

The Taqman assay successfully scored 96.6% (*n* = 247) and 94.1% (*n* = 125) of the *An. coluzzii* screened for *kdr* (L995F and L995S) and *Ace-1*^*R*^ loci, respectively. In the Sakassou mosquito population studied over 3 years, the L995F *kdr* was prevalent at frequencies ranging from 53% to 71%; in contrast, the L995S *kdr* was rare, with frequencies of between 4% and 9%. The frequency of the *Ace-1*^*R*^ mutation ranged from 38% to 51%. The L995F *kdr* showed significant variation across the study years (*χ*^2^ = 13.77,* df* = 4,* p* < 0.05), while the frequency of the L995S *kdr* (*χ*^2^ = 2.30,* df* = 4, *p* = 0.68) and the *Ace-1*^*R*^ mutation (*χ*^2^ = 7.17,* df* = 4, *p* = 0.13) were similar. Heterozygous individuals predominated at the L995F *kdr* and G280S *Ace-1*^*R*^ resistance markers, whereas homozygous individuals were more frequent at the L995S *kdr* resistance marker.

We tested the observed allelic frequencies of the target-site mutations for each study year against the HWE. In contrast to the L995S *kdr* and the G280S *Ace-1*^*R*^ mutation, the distribution of the L995F *kdr* genotypes did not follow the frequencies expected for the HWE, as shown by the *p*-value (Table 2).

**Table 2:** Knockdown and G280S acetylcholinesterase 1 resistance genotypes and associated phenotype frequencies in *Anopheles coluzzii* from Sakassou, Central Côte d’Ivoire

Mutation	Year	Genotype^a^						χ^2^	*p*-value
		RR		RS		SS			
		Observed	Expected	Observed	Expected	Observed	Expected		
L995F-*kdr*	2018	21	25.41	30	21.18	0	4.41	8.85	0.01
	2019	9	14.83	37	25.34	5	10.83	10.79	0.01
	2020	4	7.20	16	9.60	0	3.20	8.89	0.01
L995S*-kdr*	2018	0	0.18	6	5.67	45	45.13	0.05	0.03
	2019	1	0.49	8	9.02	42	41.45	0.68	0.10
	2020	0	0.04	2	1.83	21	21.06	0.05	0.48
G280S-*Ace-1*^*R*^	2018	7	6.51	11	11.98	6	5.51	0.16	0.69
	2019	4	3.76	11	11.48	9	8.76	0.04	0.84
	2020	0	1.69	9	5.63	3	4.69	4.32	0.04

The *p*-value for the Hardy–Weinberg equilibrium was calculated using the exact test procedures implemented in the R package ‘Hardy–Weinberg’ [38]

*Ace-1*^*R*^ G280S acetylcholinesterase 1 resistance mutation; *L995F-kdr*,* L995S-kdr* knockdown resistance) mutations

^a^RR: homozygote mutant; RS, heterozygote; SS, wild type

### Metabolic gene expression

**Fig. 4:**
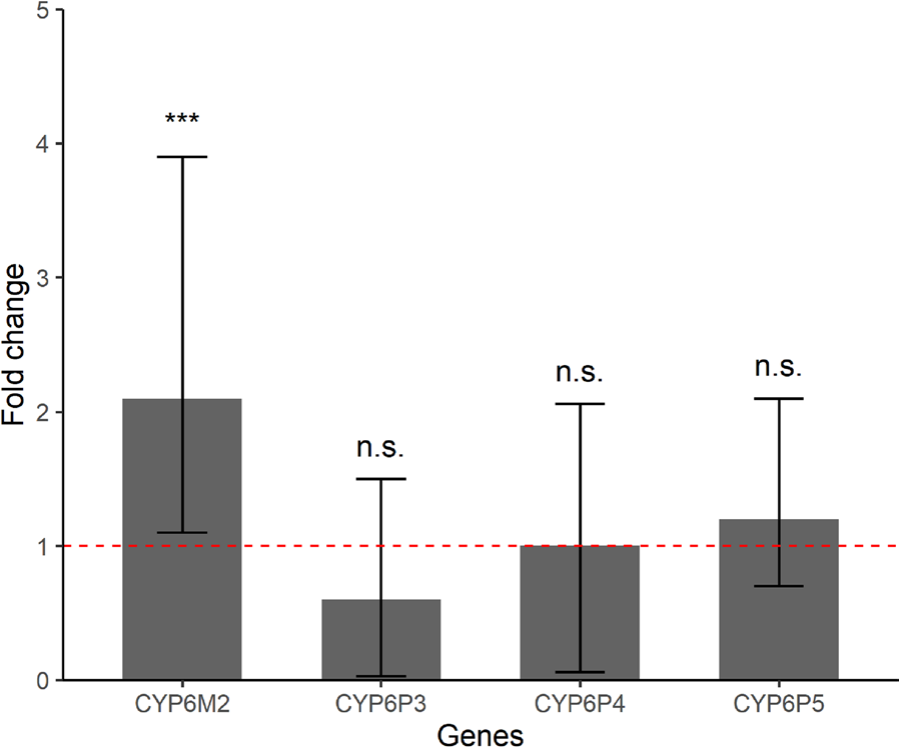
Gene expression of selected cytochrome P450 monooxygenases (CYP450s) in *Anopheles coluzzii* from Sakassou. Error bars are the 95% confidence intervals. The horizontal red dashed line represents the intercept (no difference) corresponding to the level of expression of the genes in the susceptible strain

Compared to the laboratory reference strain, CYP6M2 was overexpressed in the field-collected mosquitoes with a mean FC of 2.1 (95% CI 1.1–3.9). In contrast, CYP6P3 (FC 0.6, 95% CI 0.03–1.5), CYP6P4 (FC 1.0, 95% CI 0.06–2.1) and CYP6P5 (FC 1.2, 95% CI 0.7–2.1) were not significantly overexpressed (Fig. 4).

## Discussion

*Anopheles coluzzii* mosquitoes from Sakassou showed high levels of resistance to all the pyrethroids tested (alpha-cypermethrin, deltamethrin and permethrin), even at concentrations that were tenfold higher (10×) than the WHO diagnostic concentration, while they remained susceptible to clothianidin. Pre-exposure of the mosquitoes to PBO increased mortality when subsequently exposed to pyrethroids; however, it did not fully restore susceptibility. In addition, we found that CYP6M2 was overexpressed in the field-sampled *An. coluzzii* compared to the susceptible laboratory colony, and we detected the presence of the L995F and L995S *kdr* alleles, as well as of the G280S *Ace-1*^*R*^ resistance allele.

Many of the present results align with findings from previous studies. Indeed, *An. coluzzii* has been described as the predominant malaria vector species in Sakassou [40].

Pyrethroid resistance is widely documented across West Africa [41–43], including Côte d’Ivoire [10, 11, 44, 45]. The high level of pyrethroid resistance observed in our study is consistent with results from earlier research conducted in both Central [46] and southern Côte d’Ivoire [47–49].

Most of the *An. coluzzii* tested were heterozygous for the L995F *kdr* target-site mutation with increased frequencies over the years (2018: 58.8%, 2019: 72.5%, 2020: 80%), while the wild type allele was underrepresented. The L995F *kdr* genotypes were not in HWE, likely due to selection favouring this mutation. Agricultural activities, such as rice farming involving pesticides, particularly pyrethroids, are widely practiced in the Sakassou area and may explain the increased frequencies of the L995F *kdr* mutation observed in this study. The increased mortality observed after PBO pre-exposure in *An. coluzzii* indicates that metabolic resistance, likely involving upregulation of P450s, contributes to the phenotypic resistance to pyrethroids. Previous studies in Côte d’Ivoire have described the role of P450s in metabolic resistance in *Anopheles* vectors across the country [34, 35, 49]. Among these enzymes, CYP6M2 is well-known to confer metabolic resistance to multiple classes of insecticides, including pyrethroids [50], and it was also found to be overexpressed in the present study. Its overexpression has similarly been reported in mosquito populations from southern Côte d’Ivoire [48, 51, 52]. Our results are also consistent with findings from other West African countries, including Burkina Faso [53], Benin [50] and Ghana [54]. We also observed resistance to pirimiphos-methyl, which may be linked to the presence of the *Ace-1*^*R*^ allele [55]. This finding aligns with previous studies reporting multiple insecticide resistance mechanisms in *Anopheles* vectors across Côte d’Ivoire [52, 56]. Resistance to the organophosphate pirimiphos-methyl is particularly worrying, as it further limits the range of insecticides available for resistance management.

Intriguingly, *An. coluzzii* remained fully susceptible to clothianidin throughout the study period. Our findings are consistent with those from recent studies conducted by Kouamé et al. in Côte d’Ivoire [57] and Hougbe et al. in Benin [58], with both groups detecting susceptibility of pyrethroid-resistant *Anopheles* vectors to clothianidin. Clothianidin-based products used in IRS have demonstrated effectiveness in controlling *Anopheles* vectors in several areas with high levels of pyrethroid resistance [19, 59–61]. In Tiassalé, in southern Côte d’Ivoire, a setting with a history of high pyrethroid resistance [52, 62], a recent experimental hut trial reported that Fludora® Fusion (Bayer CropScience; Monheim, Germany) and SumiShield® (Sumitomo Chemical)—two clothianidin-based formulations—maintained residual efficacy against pyrethroid-resistant *Anopheles* vectors for up to 9 months [63]. Therefore, clothianidin could be a promising alternative active ingredient for managing pyrethroid resistance and enhance ITN control efforts in Sakassou and elsewhere in Côte d’Ivoire. While our results are promising in the context of Sakassou, reduced susceptibility to clothianidin-based products has been observed in a few other African countries. In Uganda, control failure using SumiShield^®^ 50WG was attributed to clothianidin tolerance in *Anopheles funestus* sensu lato (*An. funestus* s.l.) but not in *An. gambiae* s.l. [64]. In Cameroon, clothianidin resistance has been found in *An. gambiae* s.l. which has been attributed to cross-resistance between clothianidin and other neonicotinoids used in agriculture for crop protection [65]. In contrast, an experimental hut study, also conducted in Cameroon, showed that Fludora® Fusion, a combination of clothianidin with deltamethrin, was effective against *An. funestus* s.l. [66]

A possible limitation of the study could be the use of the *An. gambiae* s.s. Kisumu strain as the reference susceptible colony for measuring gene expression levels in *An. coluzzii*. Although these taxa are closely related sibling species, they may exhibit differences in baseline gene expression levels, potentially introducing bias in the gene expression analysis. Additionally, *An. gambiae* s.s. Kisumu is a laboratory colony that has been maintained for decades, while the *An. coluzzii* samples retain a field background; this difference could further affect comparability. Moreover, our analysis included only four P450s, leaving the possibility that we overlooked other P450s and members of other enzyme families contributing to the observed insecticide resistance phenotypes.

## Conclusions

The results of the current study revealed both target-site and metabolic resistance mechanisms associated with high intensities of pyrethroid and organophosphate resistance in the predominant malaria vector, *An. coluzzii*, in Sakassou, Central Côte d’Ivoire. This finding poses a significant challenge to the malaria vector control programmes relying solely on pyrethroid-only ITNs. Since the local mosquito population remains susceptible to clothianidin, complementary IRS applications using clothianidin-based products could be a viable alternative. However, should this strategy be adopted, we strongly recommend ongoing and regular monitoring of insecticide susceptibility, including the underlying resistance mechanisms, to inform and guide the implementation of effective malaria vector control strategies and avoid any control failure.

## Data Availability

The datasets supporting the findings are either included within the manuscript or may be provided by the corresponding author on reasonable request.

## Abbreviations

*Ace-1*^*R*^: Acetyl cholinesterase 1 insensitive allele
CYP450s: Cytochrome P450 monooxygenases
FC: Fold change
GLM: Generalised linear model
HWE: Hardy–Weinberg equilibrium
IRS: Indoor residual spraying
ITN: Insecticide-treated net
*kdr*: Knockdown resistance allele
PBO: Piperonyl butoxide
RT-qPCR: Reverse transcriptase–quantitative PCR
WHO: World Health Organization
